# Plant height shapes hydraulic architecture but does not predict metaxylem area under drought in *Sorghum bicolor*

**DOI:** 10.1002/pld3.498

**Published:** 2023-05-22

**Authors:** Melissa A. Lehrer, Jennifer S. Hawkins

**Affiliations:** ^1^ Department of Biology West Virginia University Morgantown West Virginia USA; ^2^ Department of Ecosystem Science and Management The Pennsylvania State University University Park Pennsylvania USA

**Keywords:** drought, hydraulic damage, plant morphology, *Sorghum bicolor*, stomatal aperture, transpiration

## Abstract

Climate change‐induced variations in temperature and precipitation negatively impact plant growth and development. To ensure future food quality and availability, a critical need exists to identify morphological and physiological responses that confer drought tolerance in agro‐economically important crop plants throughout all growth stages. In this study, two *Sorghum bicolor* accessions that differ in their pre‐flowering responses to drought were exposed to repeated cycles of drying and rewatering. Morphological, physiological, and histological traits were measured across both juvenile and adult developmental stages. Our results demonstrate that plant height is not predictive of metaxylem area but does influence the hydraulic path and water management in an accession‐specific manner. Further, when drought‐responsive changes to the plant architecture are unable to compensate for the hydraulic risk associated with prolonged drought exposure, tight control of stomatal aperture is crucial to further mitigate hydraulic damage and prevent xylem embolism.

## 1 INTRODUCTION

Drought is one of the main environmental constraints limiting crop yield, resulting in a 20–70% decrease in agricultural production (Dai, 2011; Datta, 2004; Fracasso et al., 2016). Due to its unpredictability and overall impact on plant growth and development, drought is a major threat to global food quality, availability, and affordability (Barnabás et al., 2007; Qadir et al., 2015; Yordanov et al., 2000; Zhu, 2016). As climate change‐induced drought is predicted to become more frequent, and in conjunction with rising temperatures, it is imperative to identify mechanisms that enhance plant resistance to water‐limited conditions (Daryanto et al., 2016; Pachauri et al., 2015). Therefore, refining the definition of drought tolerance through the characterization of morphological, histological, and physiological mechanisms that promote hydraulic safety is crucial to determine how crop plants combat the hydraulic consequences of drought exposure.

Morphological responses to drought stress in plants have been well characterized (Blum, 1996; Chaves & Oliveira, 2004; Flower et al., 1990; Moussa & Abdel‐Aziz, 2008; Ochieng et al., 2021; Tuinstra et al., 1997). Drought‐responsive shifts include reductions in height, leaf area, and aboveground biomass, which collectively work to partition assimilates belowground, minimize transpirational water loss, and/or impact hydraulic architecture (Johnson et al., 2014; Liu et al., 2014; Olson et al., 2018; Zhang et al., 2015). Additional responses that influence plant hydraulics include modifications to the vasculature, namely, the xylem. For example, as discovered in response to a long‐term acclimatization study, changes in xylem traits were found to influence hydraulic conductivity, stomatal conductance, and other whole‐plant features in *Phaseolus vulgaris* (the common bean) (Holste et al., 2006). Further, modification of xylem size and/or density (i.e., vascular bundle number) can impact hydraulic resistance, ultimately working to reduce the risk of xylem embolism (Holste et al., 2006; Tognetti et al., 1997). Belowground, changes in root length, angle, and/or root hair density enhance water acquisition from deeper soil layers (Ali et al., 2009; Borrell et al., 2014; Redillas et al., 2012; Singh et al., 2010).

Physiological responses, especially those that impact the plant's water status, also play a major role in drought resistance. For example, given the essential role of stomata in gas exchange and transpiration, modifications to stomatal aperture and density can alter a plant's water use efficiency (Bertolino et al., 2019). As described in Caine et al. (2019), drought‐stressed rice plants with a lower stomatal density were found to have cooler leaf temperatures compared with those with higher stomatal density. These findings suggest that, under stress, plants with fewer stomata per unit area were able to maintain stomatal conductance (Caine et al., 2019). Further, in response to prolonged periods of stress, external signals perceived by mature leaves can alter the stomatal development of new leaves, ultimately resulting in stress‐responsive changes to stomatal patterning (Bertolino et al., 2019). Therefore, identifying changes in these traits over developmental time can be useful in uncovering systemic responses to drought conditions (Bertolino et al., 2019).

The relationship between hydraulic architecture and morphology has been well established in woody angiosperms (Guha et al., 2018; Liu et al., 2019; Olson et al., 2018). Olson et al. (2018) found plant size to be a main driver of conduit width in a range of tree species. As such, their work emphasized the relationship between plant height, conduit width, and xylem embolism susceptibility, suggesting that taller species may be more vulnerable to cavitation events compared with shorter species (Olson et al., 2018). Despite the strong understanding of these responses in trees, the strategies used to mitigate hydraulic damage in herbaceous monocots are more obscure. Therefore, determining whether this established relationship between morphology, physiology, and hydraulic mechanisms in woody angiosperms translates into crop plants will provide a new foundation upon which to improve crop performance under abiotic stress. Additionally, much of the published work in crop plants focuses on plants exposed to short periods of drought in greenhouse settings (Akman et al., 2020; Aslam et al., 2015; Drobnitch et al., 2021; Machado & Paulsen, 2001; Moussa & Abdel‐Aziz, 2008; Munamava & Riddoch, 2001). In practice, however, individual plants can experience short periods of drought and intermittent rainfall and/or prolonged periods of drought throughout an entire growing season (Godwin & Farrona, 2020). In order to more holistically define drought tolerance, the responses to and consequences of long‐term drought exposure over developmental time require elucidation.

*Sorghum* is a staple C4 grain crop grown for food, animal feed, and biofuel. Due to its domestication in arid environments, *Sorghum* is considered to be drought tolerant. Therefore, it is an ideal model to study drought responsive mechanisms in an agriculturally and economically important grain crop. In this study, we compared the morphological, physiological, and histological responses in two *Sorghum bicolor* accessions that are known to differ in their pre‐flowering responses to drought (Premachandra et al., 1993). The impact of drought exposure on vegetative growth is well established; however, given the positive correlation between aboveground biomass and grain yield, maintaining morphological and physiological parameters following drought exposure is advantageous to prevent major grain yield losses (Casari et al., 2019). Therefore, we hypothesized that maintenance of morphological and histological traits near control levels would be observed in the pre‐flowering drought tolerant accession, TX7078, whereas significant fluctuations in physiological features were expected in BTx642.

## 2 METHODS AND MATERIALS

### 2.1 Experimental design

Two accessions of *S. bicolor*, TX7078 (PI 655990) and BTx642 (PI 656029), described as pre‐ and post‐flowering drought tolerant, respectively (Premachandra et al., 1993), were obtained from the USDA Germplasm Resources Information Network (GRIN). One hundred and forty‐four replicates of each accession were germinated in 5 cm × 5 cm × 5 cm planting plugs in Premier Pro‐Mix BX MYCO soil (Premier Tech Horticulture, Quakertown, PA, USA). Greenhouse conditions during germination were as follows: 21°C, 75% humidity, and .62‐kPa vapor pressure deficit; seedlings were misted with tap water during germination. Once seedlings reached the two‐leaf stage (23 days post sowing), they were transplanted into 5 cm × 5 cm × 25 cm tree pots (Stuewe and Sons, Tangent, OR, USA) in a 3:1 combination of #4 silica sand and Premier Pro‐Mix BX MYCO soil. Plants were organized by treatment group (control and drought stressed) and randomly sown. Conditions of the greenhouse room were as follows: 27°C/23°C (day/night), 16 h of natural and/or supplemental light, 25% humidity, and vapor pressure deficit 2.68 kPa/2.11 kPa (day/night). Following transplant, seedlings were watered with tap water every day for 1 week; plants were treated once during this establishment period with 80 ppm of 20–20–20 N–P–K (Jack's Classic Water Soluble Fertilizer, Allentown, PA, USA).

Following the establishment period, all seedlings were watered to 100% water content, as measured with a precalibrated SM150 Soil Moisture Sensor using the perlite setting (Dynamax, Houston, TX, USA). Controls were watered every day to every other day throughout the study. Drought‐stressed plants were allowed to dry to 0% water content and remained at this level for 2 days; water content was assessed every other day until 0% was reached. Upon completion of the treatment, drought‐stressed plants were watered to 100% water content; this watering regime was repeated one, two, four, and six times (henceforth referred to as cycles) to mimic periods of drought with intermittent rainfall (Table S1). Following rewatering, all plants were fertilized with 80 ppm of 20–20–20 N–P–K (Jack's Classic Water Soluble Fertilizer, Allentown, PA, USA).

### 2.2 Phenotypic measurements

Height (cm) and culm diameter (mm) were recorded at the end of each collection cycle, on all live replicates, prior to rewatering (Table S2). Height was measured from the base of the plant to the tip of the tallest leaf. Culm diameter was measured with calipers between the base of the plant and first internode.

Leaf/canopy temperature has been shown to be a useful proxy for transpiration rate and/or stomatal conductance (Blum et al., 1982; Lopes & Reynolds, 2010; Pallas et al., 1967; Wang et al., 2013; Zhang et al., 2019). As room temperature was kept constant in this study, we used leaf temperature as a measure of transpirational cooling. Leaf temperature (°C) was measured in the middle of the third newest fully expanded leaf of three to seven replicates per accession and treatment group (Table S2). Leaf temperature was recorded before watering (i.e., pre‐rewatering, approximately 10 a.m.) and then 5 min (approximately 11 a.m.), 30 min (approximately 12 p.m.), and 6 h post‐watering (approximately 4 p.m.), using the FLIR TG165 Imaging Infrared Thermometer and Thermal Camera with an emissivity setting of .95 (Pandya et al., 2013).

### 2.3 Histological analysis

Stem tissue between the base of the plant and first internode was collected from both accessions and treatment groups at 318 days post sowing (Table S1) and stored in 50% ethanol at 4°C. The day of imaging, stems were hand cut with a razor blade, submerged in dH_2_O for 3 min, and stained with .025% toluidine blue (Carolina Biological Supply Company, Burlington, NC, USA) for 5 min. Cross sections were de‐stained with dH_2_O until the water ran clear. Cross sections were mounted in 50% glycerol and imaged at 10× magnification on a compound Zeiss Observer.Z1 microscope with an Axiocam 503 Color Camera. Metaxylem diameter was measured in both the vertical (major) and horizontal (minor) axes in ImageJ; these values were used to calculate area of the metaxylem, using the following formula:

$$\text{Metaxylem Area}\;\left(\mu m^{2}\right)=\pi \times \frac{1}{2}A\times \frac{1}{2}B,$$

where A is the diameter of the major axis of the metaxylem and B is the diameter of the minor axis of the metaxylem.

To determine the number of vascular bundles per culm, the number of vascular bundles in each collected image were counted (a minimum of 10 images per replicate). Next, the diameter of each mounted stem cross section was measured; this value was used to calculate the area of the stem cross section (Area = $\pi r^{2}$, where r is equal to half the diameter of the stem cross section). Lastly, the following formula was used to calculate the number of vascular bundles per culm, where .6 mm^2^ refers to the area under the microscope:

$$\mathrm{No}.\;\text{of Vascular Bundles}\;\mathrm{Per}\;\text{Culm}=\left(\mathrm{No}.\;\text{of Vascular Bundles}\;\mathrm{Per}\;\text{Image}\right)\times \left(\left(\text{Cross Section Area,}\;{\mathrm{mm}}^{2}\right)/\left(0.6\;{\mathrm{mm}}^{2}\right)\right).$$

### 2.4 Stomatal density

In a 2019 study, where the two *S. bicolor* accession used in this work were exposed to comparable watering regimens, stomatal impressions were collected from the middle of the third newest fully expanded leaf at three time points (Table S3). Stomatal impressions were collected using clear nail varnish, and the number of stomata was counted at five locations across the impression using an Olympus CX31 compound light microscope at 40× magnification (ocular lens = 16×, field of view diameter = .25 mm). The number of stomata per 1 mm was determined using the following formula:

$$\text{Stomatal Density}\;\left(\mathrm{per}\;\mathrm{mm}\right)=\left(\text{Number of stomata}\;\mathrm{per}\;0.25\;\mathrm{mm}\right)\times 4.$$

### 2.5 Statistical analysis

Normality of the data was assessed in SAS JMP (version 14.3) and/or R (Version 4.1.0) via a Shapiro–Wilk test. Data that were not normally distributed were transformed as necessary, and these transformed values were used in downstream analyses. If data could not be normalized, a nonparametric analysis (Kruskal–Wallis test by ranks) was used to identify treatment effects (*stats* package; R Version 4.1.0; R Core Team, 2013). Otherwise, one‐way analysis of variance was performed on normalized data in SAS JMP and/or R to identify treatment effects. Significance was assessed at α = 0.05.

To identify and compare accession‐specific effects for each phenotype, the percent change from control for each phenotypic measurement was determined for both accessions, using the following formula:

$$\text{Percent Change}\;\left(\%\right)=\left(\left(\mathrm{DS}\;\text{Value}-\text{Control Average}\right)/\left(\text{Control Average}\right)\right)\times 100,$$

where Control Average refers to the average of all control values for a particular phenotypic measurement and DS Value refers to the individual value for each drought‐stressed replicate for a particular phenotypic measurement. Control Average was used to account for and minimize any minor biological differences across replicates for each accession. Percent change values were assessed via one‐way analysis of variance or Kruskal–Wallis test by ranks, using the same parameters as described above, to uncover accession‐specific responses for each phenotype across cycles. All boxplots and line plots were generated in R Version 4.1.0 using *ggplot2* (version 3.3.5; Wickham, 2016).

## 3 RESULTS

### 3.1 Plant morphology

**FIGURE 1:**
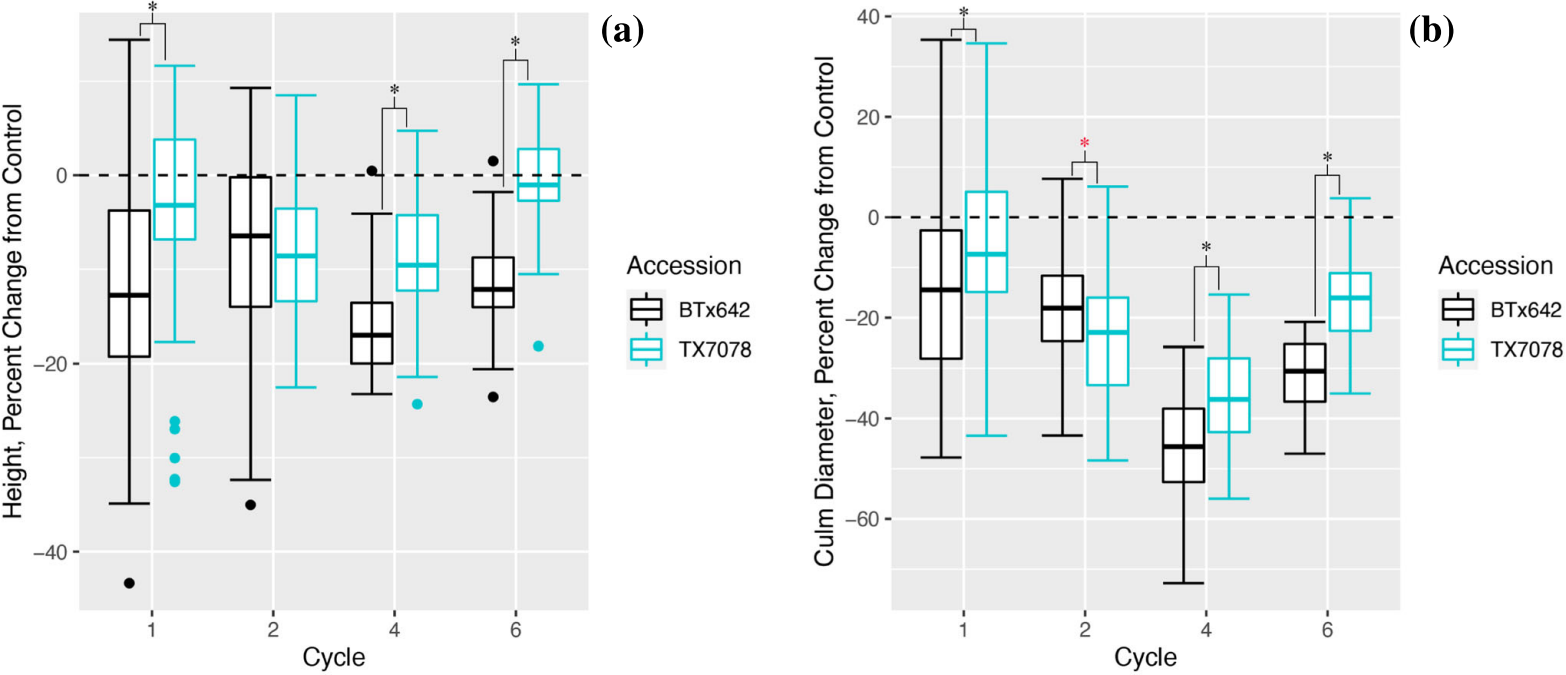
Plant morphology is maintained nearer to control levels in TX7078 compared with BTx642 following cyclical drought exposure. Following one, four, and six cycles of drought and rewatering, plant height (a) and culm diameter (b) are maintained closer to control levels in the pre‐flowering drought tolerant accession, TX7078 (teal), compared with the post‐flowering drought tolerant accession, BTx642 (black). Black asterisks indicate a significant difference between accessions, where TX7078 performed nearer to control levels compared with BTx642 (*p* < 0.05). The black dashed line at *y* = 0 reflects a 0% change from average control levels.

Plant height was reduced in response to drought in both accessions (Figures 1a and S1). A greater proportion of height was lost in the post‐flowering tolerant accession, BTx642, compared with the pre‐flowering tolerant accession, TX7078, after one (*p* < 0.0001), four (*p* = 0.0006), and six (*p* < 0.0001) cycles; there was an equal reduction in height between accessions after two cycles (*p* = 0.6209). Overall, TX7078 maintained a height more similar to controls compared with BTx642 across nearly all cycles.

Reductions in culm diameter (Figure S2) were observed in both accessions in response to prolonged drought exposure. Similar to plant height, the culm diameter of TX7078 was maintained nearer to control levels compared with BTx642 after one (*p* = 0.0010), four (*p* = 0.0008), and six (*p* < 0.0001) cycles of drought (Figure 1b).

### 3.2 Leaf temperature

**FIGURE 2:**
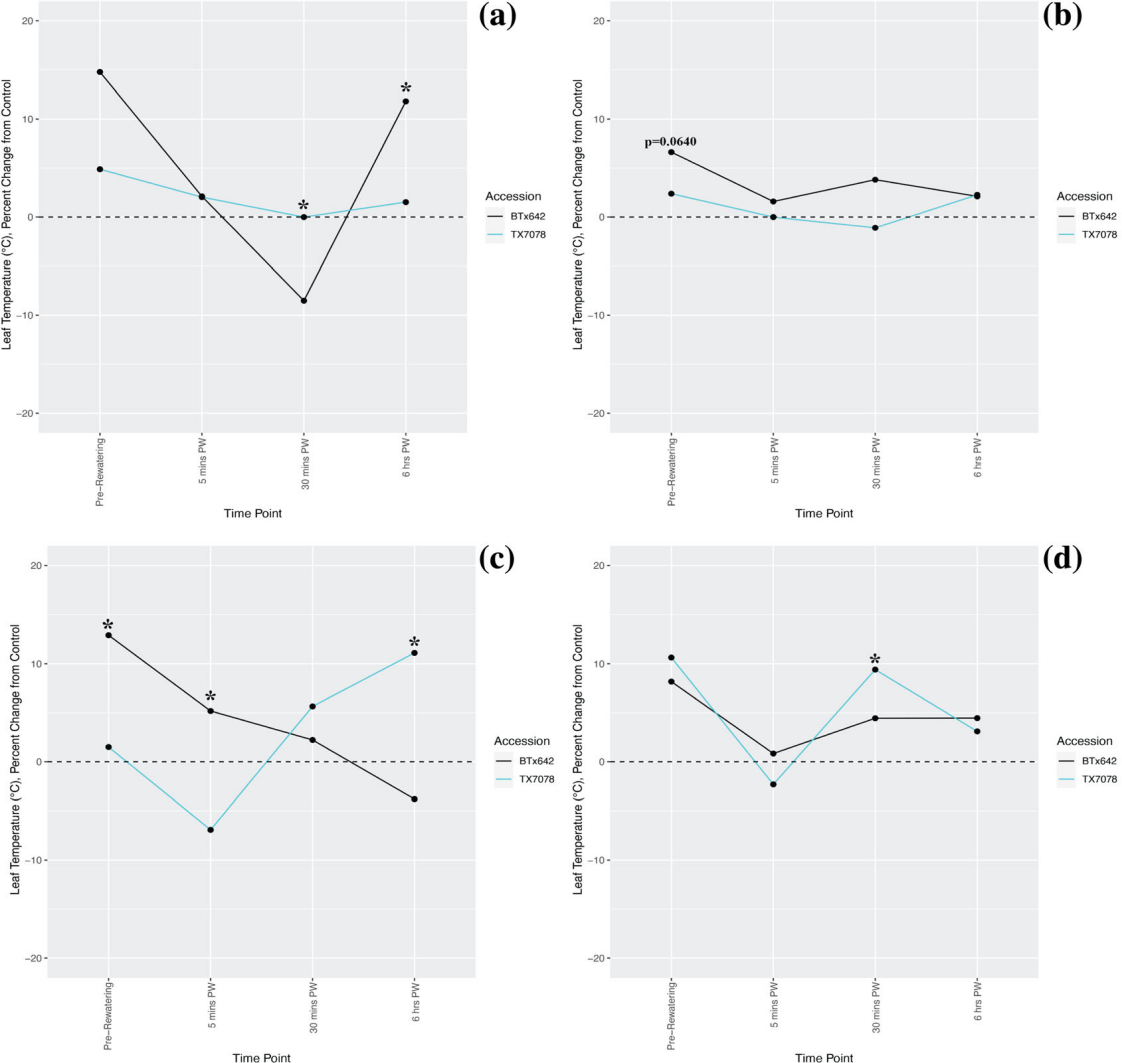
Leaf temperature is maintained nearer to control levels in TX7078 following prolonged pre‐flowering drought exposure compared with BTx642. Following cyclical drought exposure, significant fluctuations in leaf temperature were detected in BTx642 after one cycle, at the 6 h post‐rewatering (PW) time point (a). This trend persists following both two (b) and four (c) cycles of drought and rewatering, where leaf temperature is more consistently maintained closer to control levels in TX7078 compared with BTx642. However, TX7078 transitioned into the reproductive stage of development between the fourth and sixth cycles, displaying post‐flowering drought sensitivity. As such, the impact of drought exposure more prominently affected this accession after six cycles (d). The black dashed line at *y* = 0 reflects a 0% change from average control leaf temperature. Black asterisks indicate a significant difference between accession (*p* < 0.05).

After one cycle of drought, leaf temperature of TX7078 was more consistently maintained at control levels across all time points compared with BTx642; this trend was statistically significant 30 min (*p* = 0.035) and 6 h (*p* = 0.0109) post‐rewatering (Figures 2a, S3A, and S4A). Although there were no significant accession‐specific changes in leaf temperature after two cycles, the trends uncovered after one cycle are still observed: Leaf temperature is again maintained nearer to control levels in TX7078 compared with BTx642 (Figures 2b and S3B).

After four cycles of drought, maintenance of leaf temperature nearer to control levels was observed in TX7078 at the pre‐rewatering (*p* = 0.00328) and 5 min (*p* = 0.00398) post‐watering time points. However, at the 6 h post‐watering time point, this trait is increased in TX7078 compared with BTx642 (*p* = 0.013). There were no accession‐specific differences in leaf temperature uncovered at the 30 min post‐watering time point (*p* = 0.136; Figures 2c, S3C, and S4C). Lastly, following six cycles of drought and rewatering, accession‐specific changes in leaf temperature were observed 30 min post‐rewatering, where this trait was significantly increased in TX7078 compared with BTx642 (*p* = 0.0167; Figures 2d, S3D, and S4D).

### 3.3 Stomatal density

**FIGURE 3:**
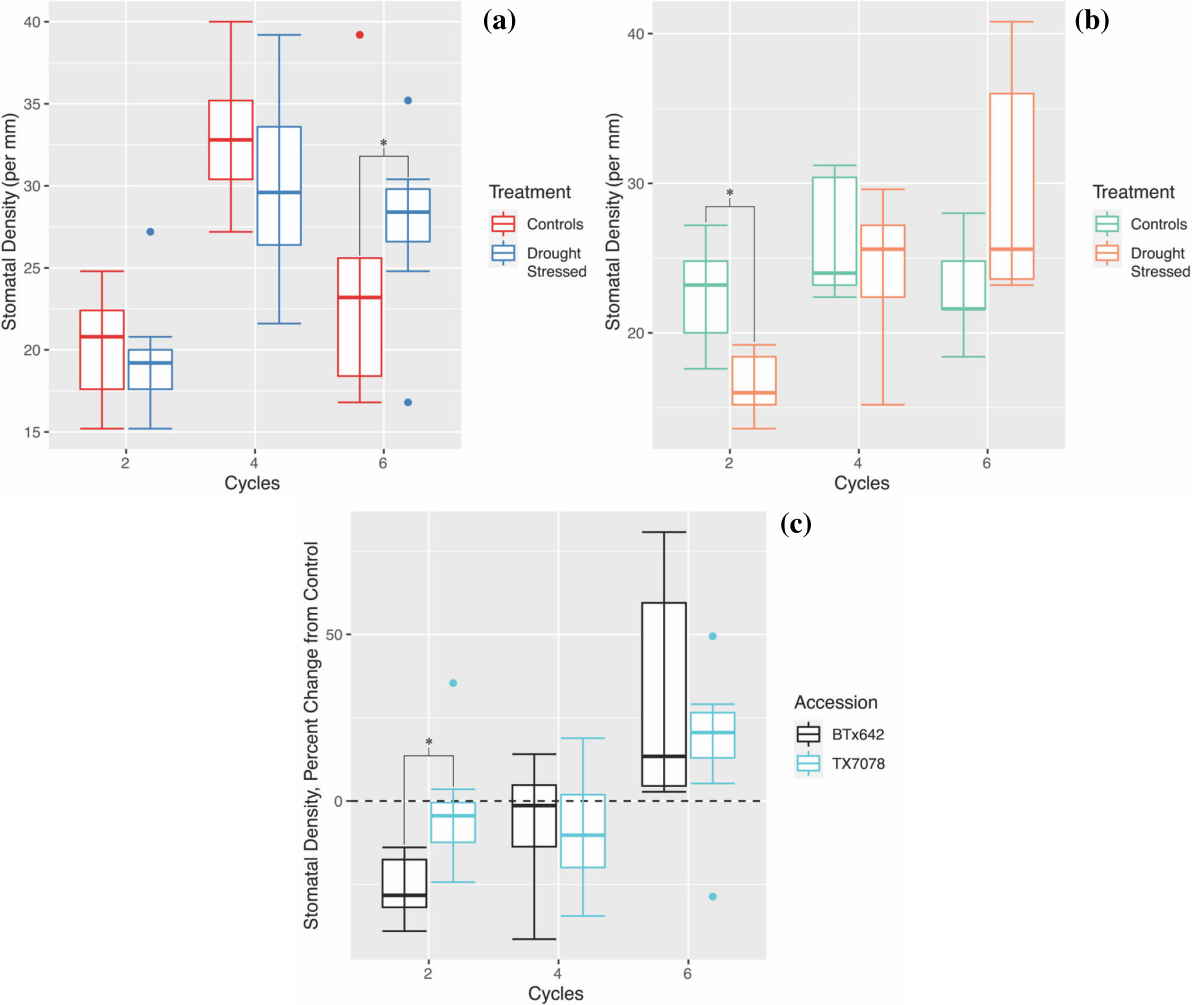
Minimal variability in stomatal density is observed in response to drought in TX7078 and BTx642. Stomatal density of TX7078 (a) was maintained at control levels after two and four cycles of drought and rewatering; however, this trait significantly increased in response to drought after six cycles. Stomatal density of BTx642 (b) is initially reduced after two cycles but is then maintained at control levels after both four and six cycles. Upon normalization to controls, stomatal density is maintained at control levels in TX7078 compared with BTx642 after two cycles. However, after four and six cycles, no accession‐specific effects on stomatal density are detected (c).

Stomatal density of TX7078 (Figure 3a) was maintained at control levels after two and four cycles of drought and rewatering (*p* = 0.706, *p* = 0.195); this trait then increased in response to drought after six cycles (*p* = 0.019). Stomatal density of BTx642 decreased in response to drought after two cycles (*p* = 0.000418) (Figure 3b) but was maintained at control levels after four and six cycles (*p* = 0.423, *p* = 0.198). There was a significant difference in stomatal density between accessions after two cycles (Figure 3c; *p* = 0.00235); however, there were no accession‐specific changes in this trait in response to drought after four and six cycles (*p* > 0.05).

### 3.4 Metaxylem area and vascular bundle number

**FIGURE 4:**
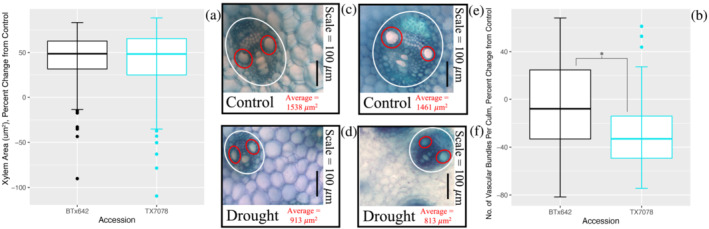
Metaxylem area is reduced in response to drought in both TX7078 and BTx642, and vascular bundle number is more variable. Metaxylem area (a) was equally reduced from control levels in response to drought in both *Sorghum bicolor* accessions. However, the number of vascular bundles per culm (b) is maintained at control levels in BTx642 (black) but is significantly reduced in TX7078 (teal) following prolonged drought exposure. Images reflect average metaxylem size for each accession ([c] TX7078, control; [d] TX7078, drought stressed; [e] BTx642, control; [f] BTx642, drought stressed). Vascular bundles are circled in white, and metaxylem are circled in red. Scale = 100 μm.

Metaxylem area was reduced in both accessions in response to repeated and prolonged drought exposure (Figure S5); however, there were no accession‐specific differences in this trait (Figure 4a; *p* = 0.696). In contrast, the number of vascular bundles per culm decreased by 29% in TX7078 in response to drought (*p* = 0.000879) but was unchanged in response to drought in BTx642 (*p* = 0.57) (Figures 4b and S6).

## 4 DISCUSSION

### 4.1 Plant height does not predict metaxylem diameter following prolonged drought exposure but may act as an early indicator of drought tolerance

Although plant size is found to be a major predictor of conduit diameter in tree species (Olson et al., 2018), we did not find this trend to be translated in these *Sorghum* accessions. Although height and culm diameter were reduced in both accessions in response to drought (Figure 1a,b), these traits were more consistently maintained near control levels in the pre‐flowering tolerant accession, TX7078. Additionally, this accession displayed a shorter stature across nearly all cycles compared with the post‐flowering tolerant accession, BTx642, under both drought and control conditions (Figure S1). Despite this observed variability in stem traits, there were no accession‐specific changes in metaxylem area (Figures 4 and S4), suggesting that, although stress responsive, this trait is fixed in these *Sorghum* accessions. Stress‐responsive decreases in metaxylem area are advantageous under drought, particularly when balancing water uptake with transpirational water loss (Guha et al., 2018; Lovisolo & Schubert, 1998; Priatama et al., 2022). The drought‐induced modifications to the vasculature identified in both accessions would act to increase hydraulic resistance within the xylem, impeding water flow within the transpiration stream and acting as a water saving mechanism (Boehm, 1893; Fichot et al., 2009; Guha et al., 2018; Hacke et al., 2006; Hargrave et al., 1994).

The trends in plant height and culm diameter uncovered in this work (Figure 1a,b), particularly at the two‐cycle time point, reveal that a greater proportion of height and culm width is lost in TX7078 compared with BTx642 during earlier developmental stages. TX7078 maintains an overall shorter stature, under both control and drought conditions, compared with BTx642 and displays pre‐flowering drought tolerance. These findings suggest that repeated drought exposure induces early signals of growth hindrance and may trigger a persistent response throughout the plant's vegetative growth cycle, resulting in shorter stature (e.g., in maize and sorghum; van Oosterom et al., 2021). This restriction in growth may be an early indication of drought tolerance and could be a result of plant stress memory (Baluska et al., 2018; Bruce et al., 2007; Crisp et al., 2016; Fleta‐Soriano & Munné‐Bosch, 2016; Mantoan et al., 2020; Ogle et al., 2015; van Oosterom et al., 2021; Walter et al., 2011). In other words, stress‐responsive changes in plant structure/morphology in TX7078 may be established following the first cycle of drought and rewatering and may ultimately facilitate a more efficient response to future stress exposure (Fleta‐Soriano & Munné‐Bosch, 2016; Mantoan et al., 2020).

### 4.2 Accession‐specific stomatal sensitivity plays an essential role in reducing the risk of hydraulic damage under drought

The maintenance of pre‐flowering leaf temperature at control levels was more consistently observed in the pre‐flowering tolerant accession, TX7078 (Figures 2 and S3). Fluctuations in transpiration rate in BTx642 (Figures 2 and S4) were detected either at the peak of water scarcity (pre‐rewatering) and/or during hotter times of the day (30 min post‐watering, approximately 12 p.m., and/or 6 h post‐watering, approximately 4 p.m.) in response to drought, likely to minimize transpirational water loss (Guha et al., 2018; Priatama et al., 2022; Tang & Boyer, 2008). Further, increases in leaf temperature over developmental time (i.e., across cycles) were a result of the drought stress exposure, not senescence, particularly following the first, second, and fourth cycles, before TX7078 transitioned into the reproductive stage of development; BTx642 made this transition well after the sixth cycle. Therefore, the increases in leaf temperature observed in later cycles (Figures S3 and S4) are indicative of drought‐induced stomatal closure (e.g., reduced evaporative cooling), a mechanism employed to prevent transpirational water loss under drought conditions (Guha et al., 2018; Priatama et al., 2022; Tang & Boyer, 2008).

Despite the possibility that plants lacked full turgidity at the 5 and 30 min post‐watering time points, leaf temperature findings at the 6 h post‐watering time point are indicative of the stomatal/transpirational response to drought exposure. The identification of differential, accession‐specific modifications to leaf temperature at two earlier post‐rewatering time points, however, indicates that stomatal responses are quite rapid. Therefore, the lack of accession‐specific modifications in stomatal density (Figure 3a–c), in combination with stomatal sensitivity, is likely responsible for the between‐accession variability in transpiration rate. Similar control of stomatal aperture in response to drought has been observed in maize and other sorghum accessions (Cochard, 2002; Guha et al., 2018). The early and late post‐watering control of stomatal aperture uncovered here provides (i) additional justification for the essential role of stomatal sensitivity in the drought response in grain crops and (ii) support for the further study of early/rapid responses to drought and rewatering at both the physiological and molecular levels.

The tight control of stomatal aperture, coupled with the observed reductions in metaxylem area, is critical for BTx642. This accession's taller stature and subsequently longer hydraulic path are associated with greater tension on water within the xylem, raising the susceptibility to cavitation events (Domec et al., 2008; Lechthaler et al., 2020; Liu et al., 2019; Tang & Boyer, 2008). Given that plants with wider vessels are found to be more susceptible to embolism, the identified reductions in metaxylem area would ordinarily function to reduce this risk (Haworth et al., 2017; Olson et al., 2018); however, this trait was equally reduced in both accessions. Thus, it is unlikely that metaxylem modifications alone were sufficient to prevent the occurrence of embolism in BTx642. Given this insufficiency, drought‐responsive stomatal closure in BTx642 is essential, as it increases embolism resistance by interrupting conductance between the roots and the shoots (Guha et al., 2018; Tang & Boyer, 2008). Additionally, the maintenance of vascular bundle number at control levels in BTx642 (Figures 4b and S6B) ensures the availability of usable xylem in the event that embolism formation renders some vessels nonfunctional (Tang & Boyer, 2008).

In contrast, the morphological and histological features of TX7078 do not require additional physiological adjustment and ultimately facilitate hydraulic safety. Therefore, the maintenance of pre‐flowering leaf temperature (Figures 2 and S3) and reduction of vascular bundle number (Figures 4b and S6A) indicate that the shorter stature and hydraulic path of TX7078 make it inherently less prone to hydraulic damage compared with BTx642.

Our work highlights the critical role of physiological adjustment in both reducing transpirational water loss and the risk of xylem embolism. This physiological approach is particularly crucial when the plant's hydraulic architecture is unable to compensate for the hydraulic consequences of long‐term drought exposure.

## 5 CONCLUSIONS

Our findings demonstrate how pre‐ and post‐flowering drought tolerant accessions of *S. bicolor* use morphological, histological, and/or physiological mechanisms, independently, to mitigate drought‐induced hydraulic damage over developmental time. As such, our findings (i) describe how the relationship between plant size and conduit width observed in woody angiosperms is not translatable to these *Sorghum* accessions and (ii) refine the definition of drought tolerance in *Sorghum* and further specify traits to be used for crop improvement.

## AUTHOR CONTRIBUTIONS

**Melissa A. Lehrer and Jennifer S. Hawkins:** Conceptualization; methodology; writing—review and editing. **Melissa A. Lehrer:** Investigation; curation; formal analysis; visualization and writing—original data preparation.

## CONFLICT OF INTEREST STATEMENT

MAL and JSH declare that there is no conflict of interest.

## Supporting information


**Table S1:** **Days post sowing and leaf stage for each of the five collection cycles from the 2021 study.** Biological replication for the stem collection is provided for each accession and treatment group (when applicable).
**Table S2: Sample size for morphological and physiological measurements.** Biological replication is provided for each cycle, grouped by accession and treatment group.
**Table S3: Days post sowing for each of the three collection cycles from the 2019 study.** Biological replication for each stomatal impression is provided for each accession and treatment group.

**Figure S1:** **Plant height is reduced in both TX7078 and BTx642 following cyclical drought exposure.** After only one, two, and four cycles of drought and rewatering, height was reduced in TX7078; there was no significant decrease in height after six cycles (**A**). Height was significantly reduced in BTx642 in response to drought after all cycles (**B**).

**Figure S2:** **Repeated and prolonged drought exposure decreases culm diameter in both TX7078 and BTx642.** After two, four, and six cycles of drought and rewatering, culm diameter was reduced in TX7078; there was no significant reduction in culm diameter after one cycle (**A**). Culm diameter was reduced in BTx642 after all cycles (**B**).

**Figure S3:** **Leaf temperature is often maintained at control levels following pre‐flowering drought exposure in TX7078.** Leaf temperature of the third newest fully expanded leaf was recorded pre‐rewatering, and five minutes post‐watering, thirty minutes post‐watering, and six hours post‐watering following one (**A**), two (**B**), four (**C**), and six (**D**) cycles of drought. Prior to the sixth cycle, when plants transitioned into the reproductive stage of development, leaf temperature was frequently maintained at control levels in TX7078. Black asterisks indicate significant differences between control and treatment groups (p < 0.05).

**Figure S4:** **Leaf temperature is increased at the peak of drought stress and/or during the hottest time(s) of the day in BTx642.** Leaf temperature of the third newest fully expanded leaf was recorded pre‐rewatering, and five minutes post‐watering, thirty minutes post‐watering, and six hours post‐watering following one (**A**), two (**B**), four (**C**), and six (**D**) cycles of drought and rewatering. Black asterisks indicate significant differences between control and treatment groups (p < 0.05).

**Figure S5:** **Prolonged drought exposure reduces metaxylem area in TX7078 and BTx642**. Following cyclical drought exposure, reductions in metaxylem area were detected in both TX7078 (**A**) and BTx642 (**B**). This trait was measured at the end of the study, 318 days post sowing.

**Figure S6:** **Vascular bundle number is reduced in TX7078 and maintained at control levels in BTx642 following cyclical drought exposure**. Following cyclical drought exposure, reductions in vascular bundle number were detected in TX7078 (**A**); however, this trait was unchanged compared to controls in drought‐stressed BTx642 (**B**). This trait was measured at the end of the study, 318 days post sowing.

**Data S1.** Supporting Information

## Data Availability

The data that support the findings of this study are available in the supporting information.
