# Supplementary material for: Plant height shapes hydraulic architecture but does not predict metaxylem area under drought in Sorghum bicolor
Source: Plant Direct. 2023 May 22;7(5):e498. doi: 10.1002/pld3.498 (PMC10203038; doi:10.1002/pld3.498)
Supplement: Supplementary file 1 — Table S1: Days post sowing and leaf stage for each of the five collection cycles from the 2021 study. Biological replication for the stem collection is provided for each accession and treatment group (when applicable). Table S2: Sample size for morphological and physiological measurements. Biological replication is provided for each cycle, grouped by accession and treatment group. Table S3: Days post sowing for each of the three collection cycles from the 2019 study. Biological replication for each stomatal impression is provided for each accession and treatment group. [file PLD3-7-e498-s003.docx]

**Tables**

**Supplementary Table S1: Days post sowing and leaf stage for each of the five collection cycles from the 2021 study.** Biological replication for the stem collection is provided for each accession and treatment group (when applicable).

| **Collection Cycle (2021)** | **Days Post Sowing** | **Leaf Stage (Main Stem)** | **Sample Size for Stem Collection** |
| --- | --- | --- | --- |
| 1 Cycle | 46 | 4-5 | N/A |
| 2 Cycles | 62 | 6-7 | N/A |
| 4 Cycles | 87 | 8-9 | N/A |
| 6 Cycles | 108 | 11-13 | N/A |
| Stem Collection | 318 | 13-17 | TX7078 = 3 per treatment group  BTx642 = 2-3 per treatment group |

**Supplementary Table S2: Sample size for morphological and physiological measurements.** Biological replication is provided for each cycle, grouped by accession and treatment group.

| **Collection Cycle (2021)** | **Sample Size for Height** | **Sample Size for Culm Diameter** | **Sample Size for Leaf Temperature** |
| --- | --- | --- | --- |
| 1 Cycle | TX7078 = 72 per treatment group  BTx842 = 72 per treatment group | TX7078 = 72 per treatment group  BTx842 = 72 per treatment group | TX7078 and BTx642= 3 per time point and treatment group |
| 2 Cycles | TX7078 = 48 per treatment group  BTx842 = 48 per treatment group | TX7078 = 48 per treatment group  BTx842 = 48 per treatment group | TX7078 and BTx642= 3 per time point and treatment group |
| 4 Cycles | TX7078 = 24-36 per treatment group  BTx842 = 26-36 per treatment group | TX7078 = 24-36 per treatment group  BTx842 = 26-36 per treatment group | TX7078 and BTx642= 3 per time point and treatment group |
| 6 Cycles | TX7078 = 23-24 per treatment group  BTx842 = 15-24 per treatment group | TX7078 = 23-24 per treatment group  BTx842 = 15-24 per treatment group | TX7078 and BTx642= 6-7 per time point and treatment group |

**Supplementary Table S3: Days post sowing for each of the three collection cycles from the 2019 study.** Biological replication for each stomatal impression is provided for each accession and treatment group.

| **Collection Cycle (2019)** | **Days Post Sowing** | **Sample Size for Stomatal**  **Impression Collection** |
| --- | --- | --- |
| 2 Cycles | 45 | TX7078 = 9 per treatment group  BTx642 = 9 per treatment group |
| 4 Cycles | 66 | TX7078 = 9 per treatment group  BTx642 = 9 per treatment group |
| 6 Cycles | 94 | TX7078 = 8-9 per treatment group  BTx642 = 7-9 per treatment group |
