# Supplementary figures and images for: Plant height shapes hydraulic architecture but does not predict metaxylem area under drought in Sorghum bicolor
Source: Plant Direct. 2023 May 22;7(5):e498. doi: 10.1002/pld3.498 (PMC10203038; doi:10.1002/pld3.498)

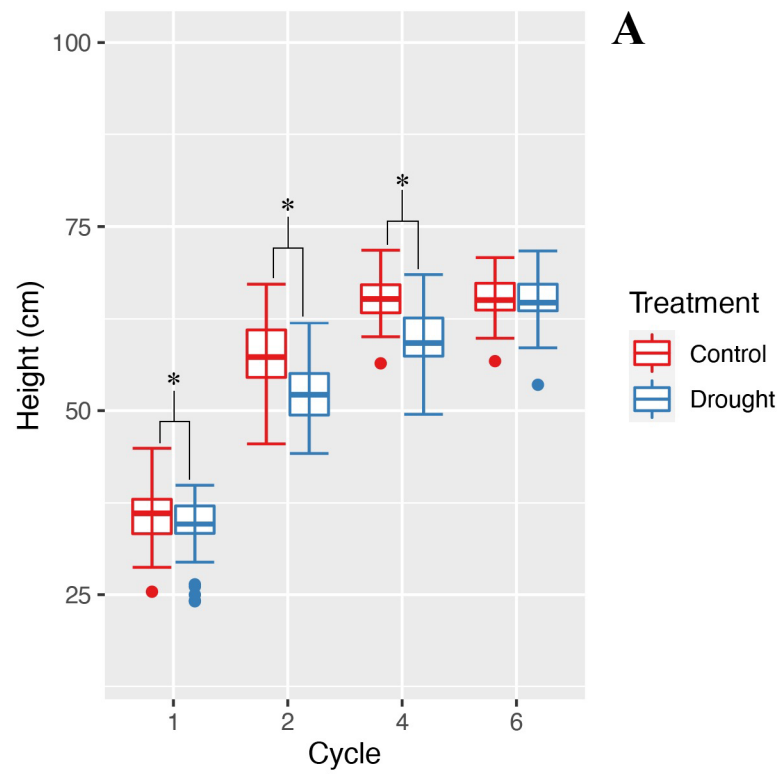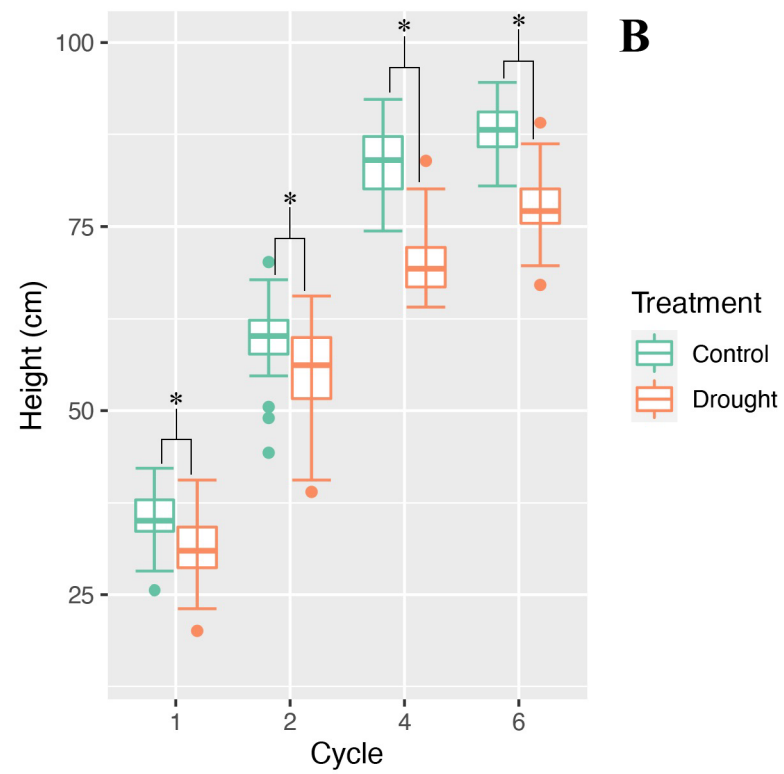

Supplement: Supplementary file 2 — Figure S1: Plant height is reduced in both TX7078 and BTx642 following cyclical drought exposure. After only one, two, and four cycles of drought and rewatering, height was reduced in TX7078; there was no significant decrease in height after six cycles (A). Height was significantly reduced in BTx642 in response to drought after all cycles (B). [file PLD3-7-e498-s004.pdf]

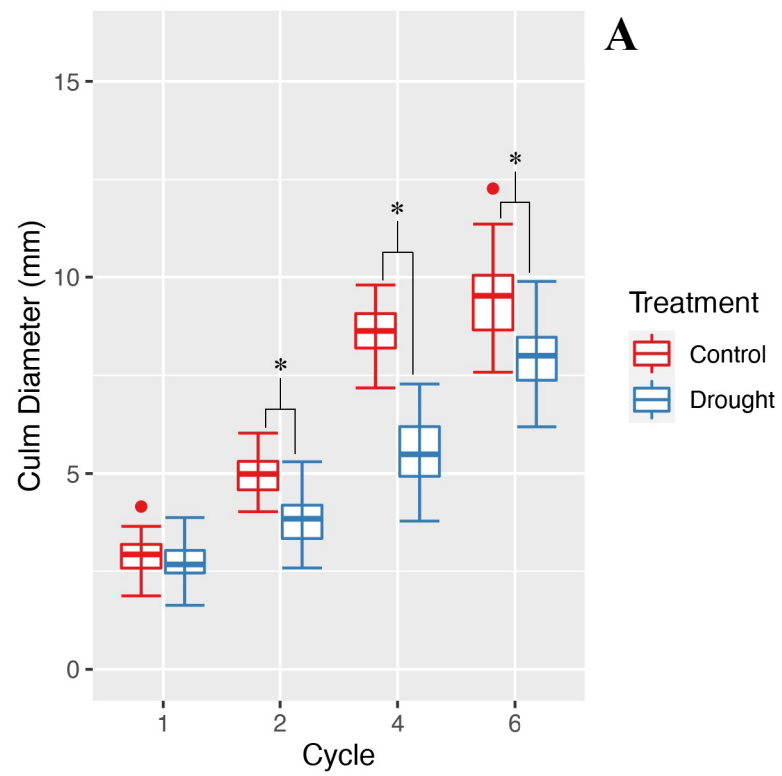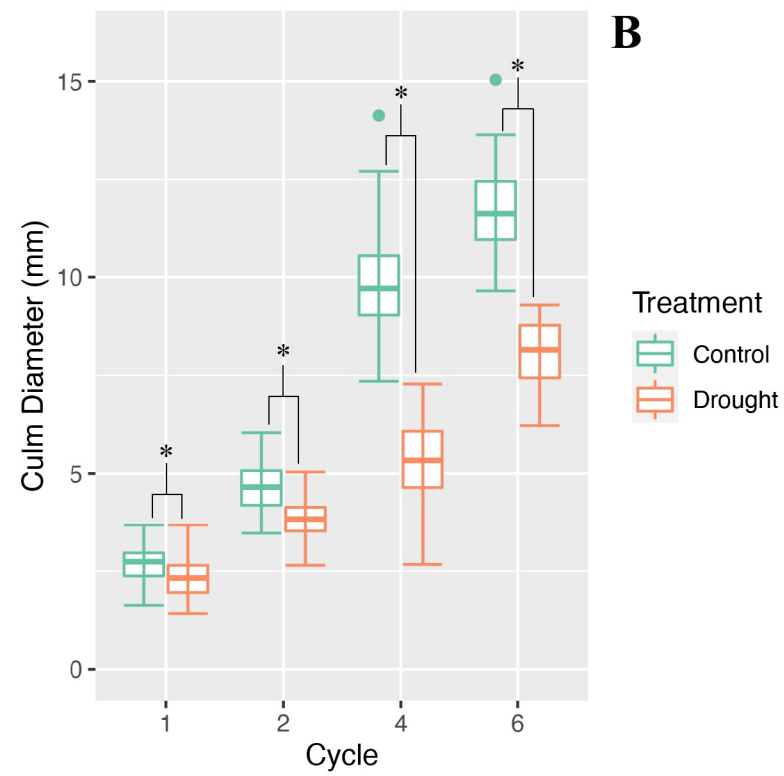

Supplement: Supplementary file 3 — Figure S2: Repeated and prolonged drought exposure decreases culm diameter in both TX7078 and BTx642. After two, four, and six cycles of drought and rewatering, culm diameter was reduced in TX7078; there was no significant reduction in culm diameter after one cycle (A). Culm diameter was reduced in BTx642 after all cycles (B). [file PLD3-7-e498-s008.pdf]

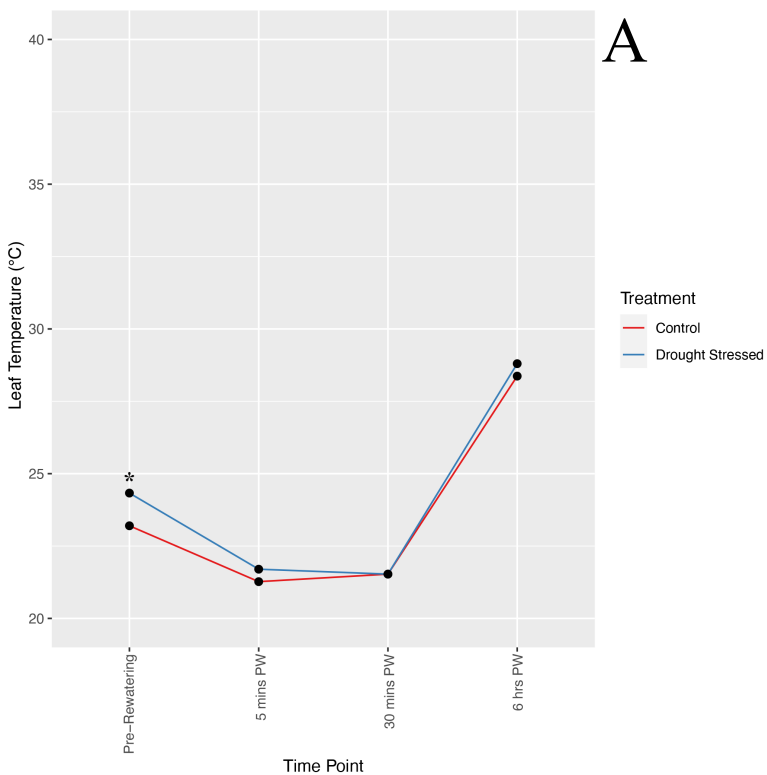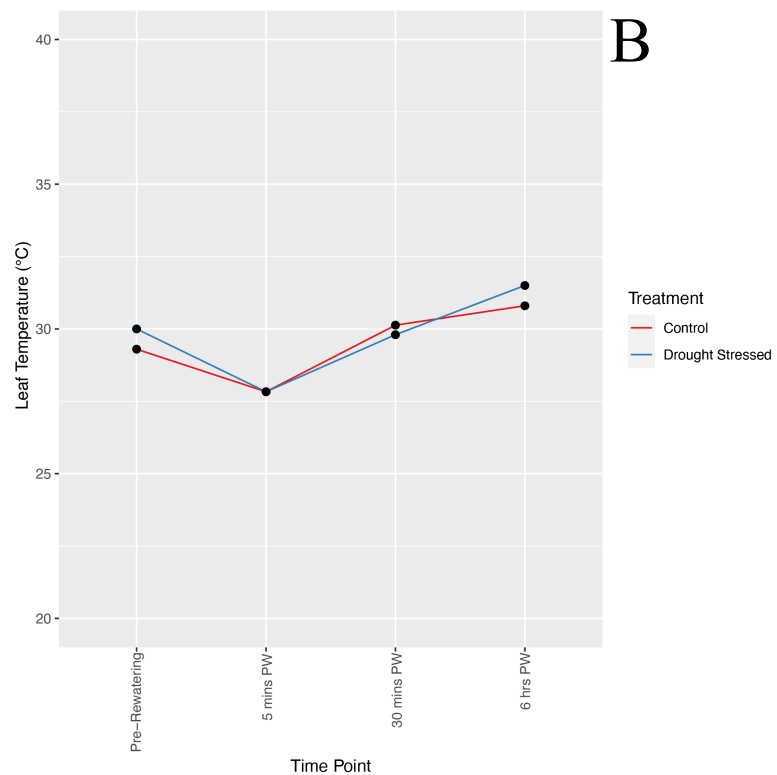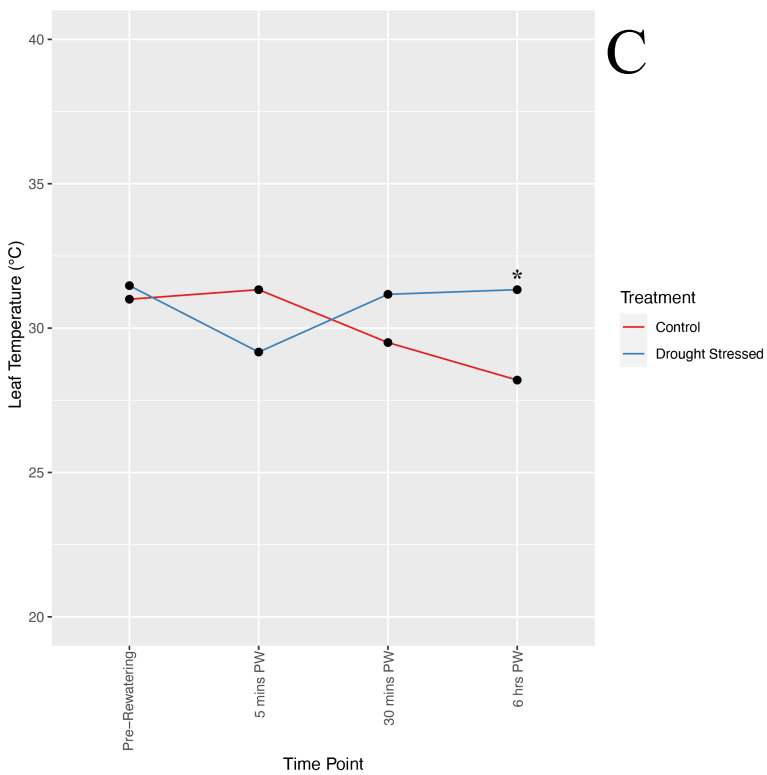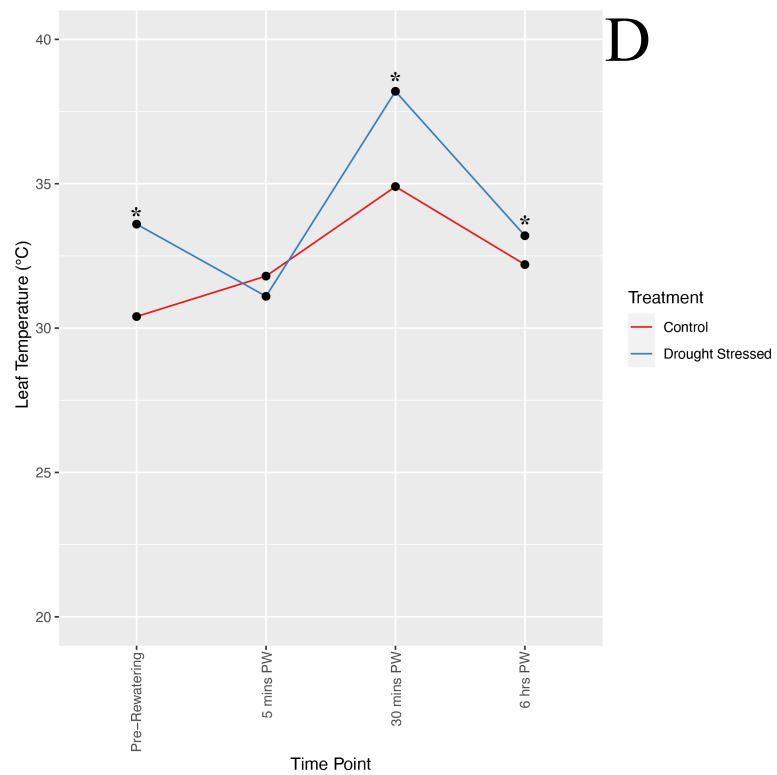

Supplement: Supplementary file 4 — Figure S3: Leaf temperature is often maintained at control levels following pre‐flowering drought exposure in TX7078. Leaf temperature of the third newest fully expanded leaf was recorded pre‐rewatering, and five minutes post‐watering, thirty minutes post‐watering, and six hours post‐watering following one (A), two (B), four (C), and six (D) cycles of drought. Prior to the sixth cycle, when plants transitioned into the reproductive stage of development, leaf temperature was frequently maintained at control levels in TX7078. Black asterisks indicate significant differences between control and treatment groups (p < 0.05). [file PLD3-7-e498-s006.pdf]

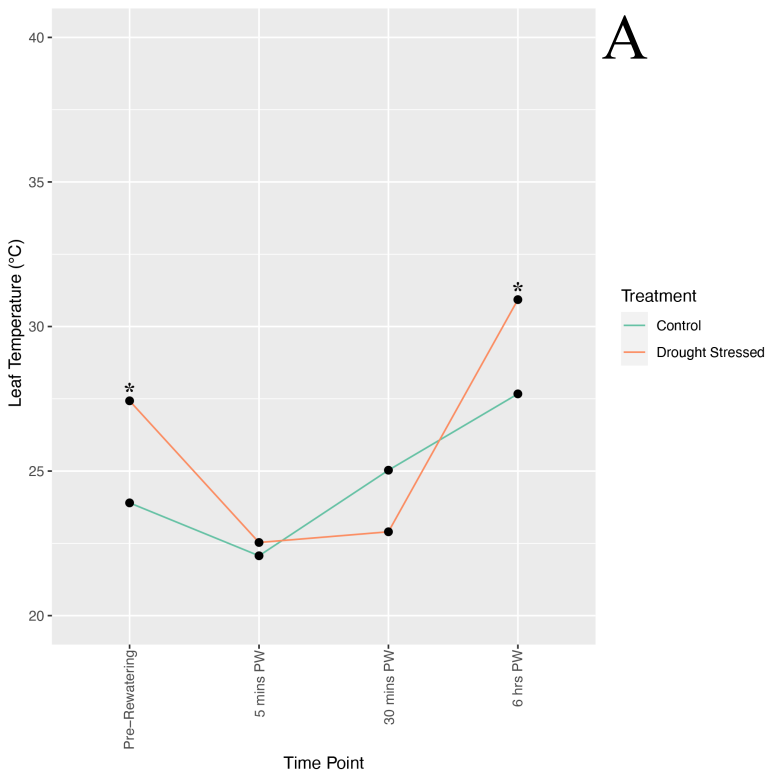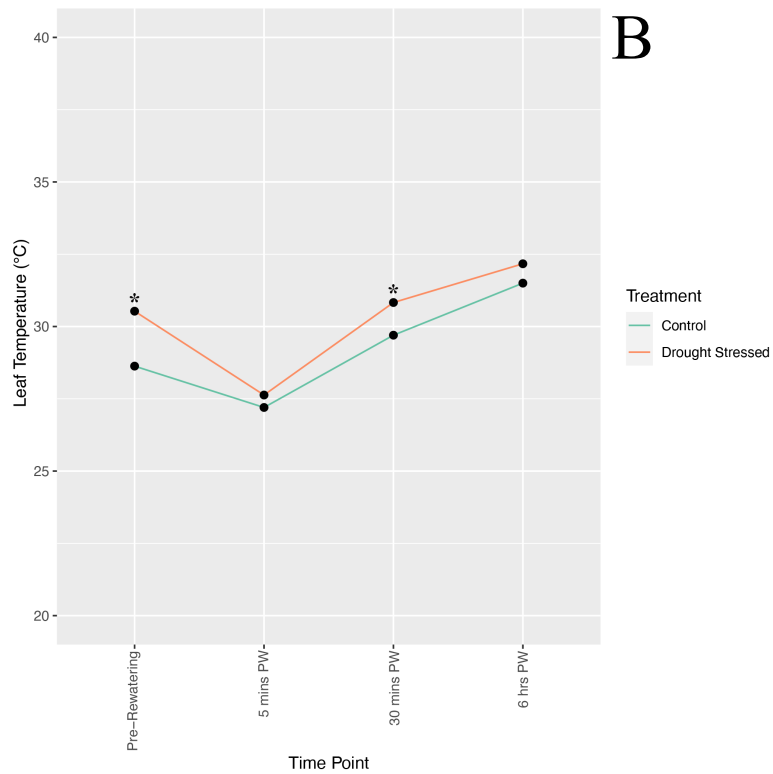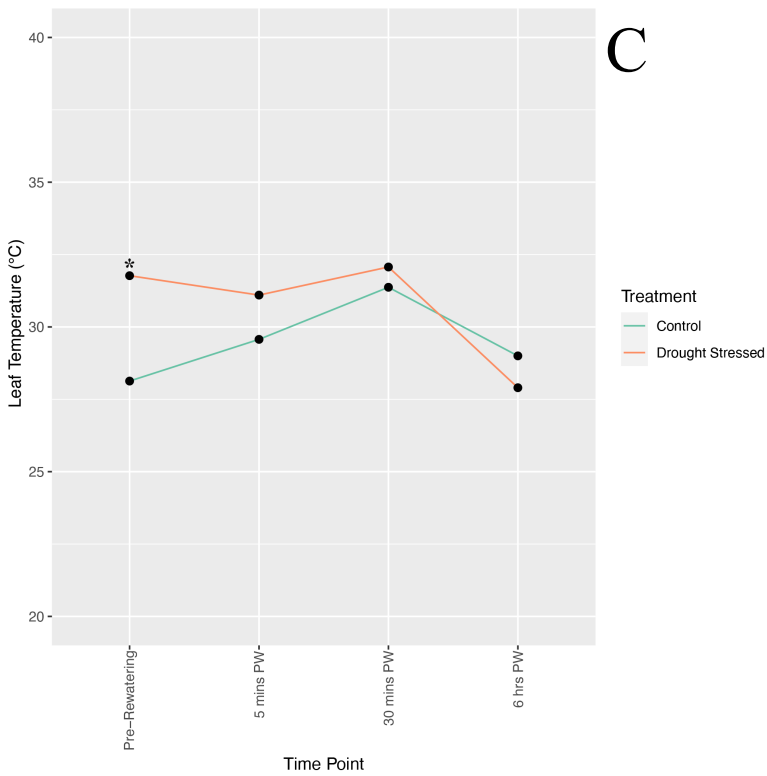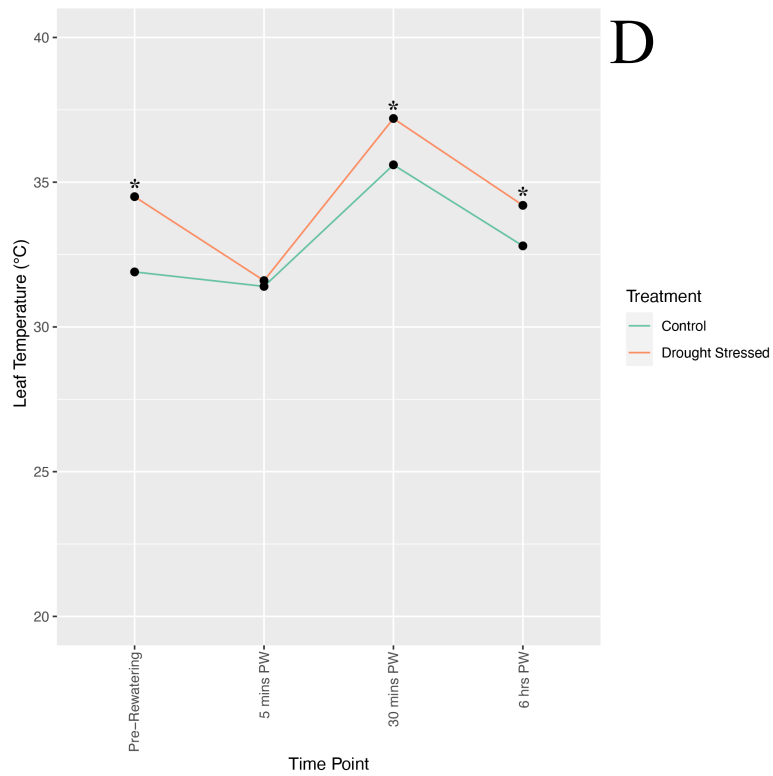

Supplement: Supplementary file 5 — Figure S4: Leaf temperature is increased at the peak of drought stress and/or during the hottest time(s) of the day in BTx642. Leaf temperature of the third newest fully expanded leaf was recorded pre‐rewatering, and five minutes post‐watering, thirty minutes post‐watering, and six hours post‐watering following one (A), two (B), four (C), and six (D) cycles of drought and rewatering. Black asterisks indicate significant differences between control and treatment groups (p < 0.05). [file PLD3-7-e498-s002.pdf]

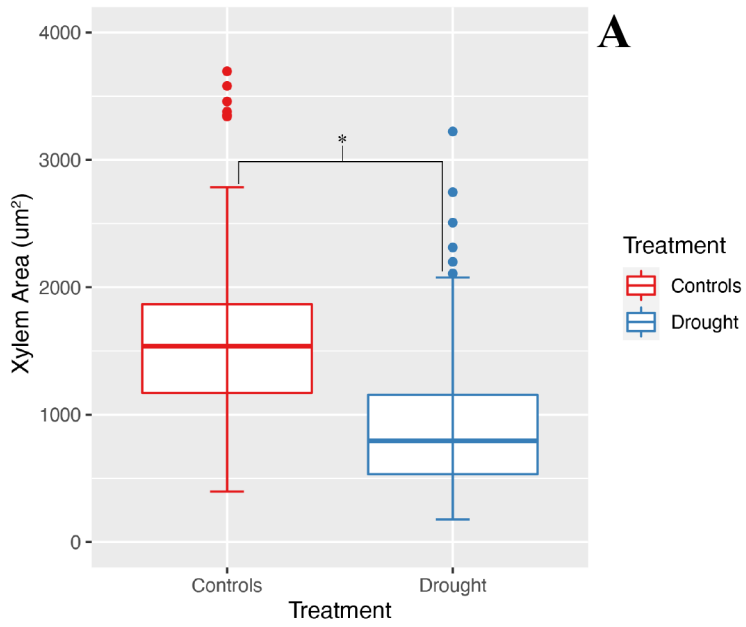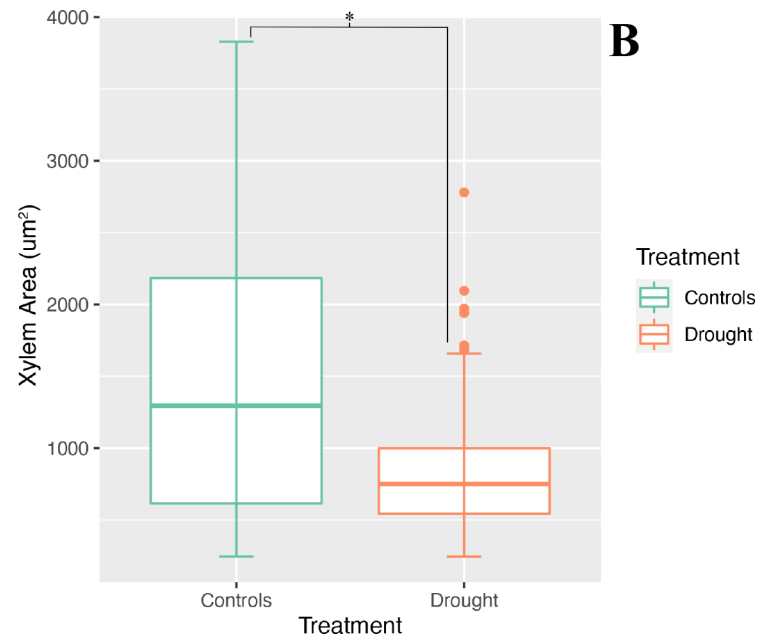

Supplement: Supplementary file 6 — Figure S5: Prolonged drought exposure reduces metaxylem area in TX7078 and BTx642. Following cyclical drought exposure, reductions in metaxylem area were detected in both TX7078 (A) and BTx642 (B). This trait was measured at the end of the study, 318 days post sowing. [file PLD3-7-e498-s001.pdf]

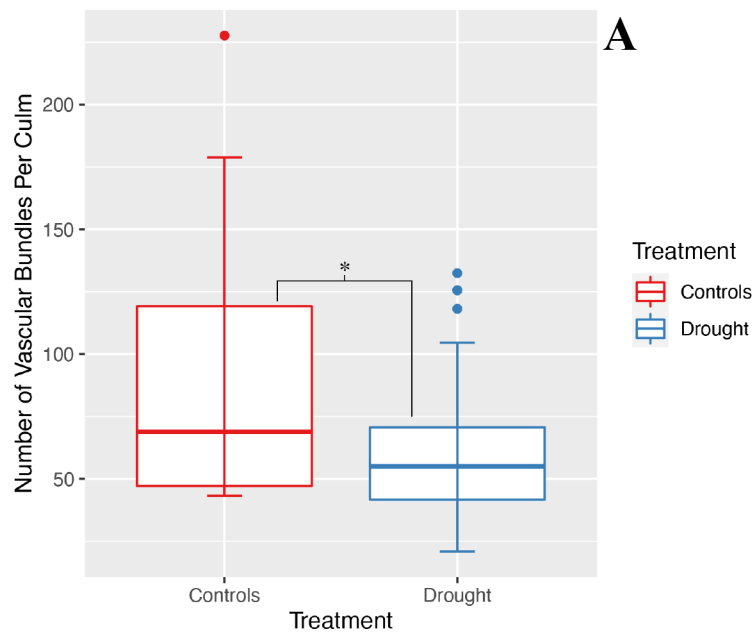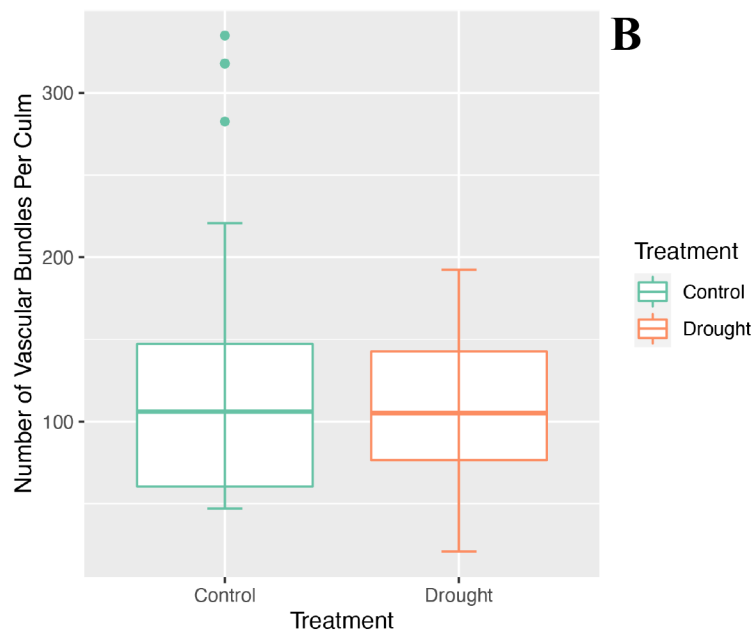

Supplement: Supplementary file 7 — Figure S6: Vascular bundle number is reduced in TX7078 and maintained at control levels in BTx642 following cyclical drought exposure. Following cyclical drought exposure, reductions in vascular bundle number were detected in TX7078 (A); however, this trait was unchanged compared to controls in drought‐stressed BTx642 (B). This trait was measured at the end of the study, 318 days post sowing. [file PLD3-7-e498-s007.pdf]
